# Sulfide mineral-driven photon upconversion sustains phototrophic cyanobacteria at deep-sea hydrothermal vents: a reply to Mullineaux and Duffy's chemolithoautotrophic hypothesis

**DOI:** 10.1093/nsr/nwaf292

**Published:** 2025-07-21

**Authors:** Yan Li, Jiaqi Zhu, Qi Li, Hao Hong, Tao Li, Haoning Jia, Bingxu Hou, Houze Lu, Yanzhang Li, Jin Xie, Fuchen Wang, Huan Ye, Kaihui Liu, Anhuai Lu, Jindong Zhao

**Affiliations:** SKLab-DeepMinE, MOEKLab-OBCE, School of Earth and Space Sciences, Peking University, China; Beijing Key Laboratory of Mineral Environmental Function, School of Earth and Space Sciences, Peking University, China; SKLab-DeepMinE, MOEKLab-OBCE, School of Earth and Space Sciences, Peking University, China; Beijing Key Laboratory of Mineral Environmental Function, School of Earth and Space Sciences, Peking University, China; Key Laboratory of Algal Biology, Institute of Hydrobiology, Chinese Academy of Sciences, China; State Key Laboratory for Mesoscopic Physics, Frontiers Science Centre for Nano-optoelectronics, School of Physics, Peking University, China; Key Laboratory of Algal Biology, Institute of Hydrobiology, Chinese Academy of Sciences, China; SKLab-DeepMinE, MOEKLab-OBCE, School of Earth and Space Sciences, Peking University, China; Beijing Key Laboratory of Mineral Environmental Function, School of Earth and Space Sciences, Peking University, China; SKLab-DeepMinE, MOEKLab-OBCE, School of Earth and Space Sciences, Peking University, China; Beijing Key Laboratory of Mineral Environmental Function, School of Earth and Space Sciences, Peking University, China; SKLab-DeepMinE, MOEKLab-OBCE, School of Earth and Space Sciences, Peking University, China; Beijing Key Laboratory of Mineral Environmental Function, School of Earth and Space Sciences, Peking University, China; SKLab-DeepMinE, MOEKLab-OBCE, School of Earth and Space Sciences, Peking University, China; Beijing Key Laboratory of Mineral Environmental Function, School of Earth and Space Sciences, Peking University, China; State Key Laboratory for Mesoscopic Physics, Frontiers Science Centre for Nano-optoelectronics, School of Physics, Peking University, China; International Centre for Quantum Materials, Collaborative Innovation Centre of Quantum Matter, Peking University, China; Key Laboratory of Algal Biology, Institute of Hydrobiology, Chinese Academy of Sciences, China; SKLab-DeepMinE, MOEKLab-OBCE, School of Earth and Space Sciences, Peking University, China; Beijing Key Laboratory of Mineral Environmental Function, School of Earth and Space Sciences, Peking University, China; State Key Laboratory for Mesoscopic Physics, Frontiers Science Centre for Nano-optoelectronics, School of Physics, Peking University, China; International Centre for Quantum Materials, Collaborative Innovation Centre of Quantum Matter, Peking University, China; SKLab-DeepMinE, MOEKLab-OBCE, School of Earth and Space Sciences, Peking University, China; Beijing Key Laboratory of Mineral Environmental Function, School of Earth and Space Sciences, Peking University, China; Key Laboratory of Algal Biology, Institute of Hydrobiology, Chinese Academy of Sciences, China; State Key Laboratory of Gene Function and Modulation Research, School of Life Sciences, Peking University, China

Our work reveals that frequency-doubling upconversion in sulfide minerals, particularly chalcopyrite, can generate visible light in deep-sea hydrothermal environments. Spectroscopic measurements demonstrate that this mechanism produces blue–green photon fluxes that are three orders of magnitude higher than the intrinsic thermal radiation from vents. To our knowledge, this represents the only natural mechanism capable of converting higher-density infrared photons into biologically relevant visible photons at deep-sea hydrothermal vents, significantly amplifying the native visible photon pool there.

Despite these findings, quantifying absolute visible photon fluxes at deep-sea hydrothermal vents remains challenging due to several factors. First, the scarcity of *in situ* spectral measurements and published data introduces significant uncertainty in determining the original photon flux at hydrothermal vents. This constraint only permits relative comparisons in current analyses, such as the deviations of visible photon flux from blackbody radiation and flux ratios across different wavelength bands, rather than absolute quantification. Second, our current measurements of mineral upconversion efficiency likely represent conservative estimates for natural systems. This limitation arises because the efficiency quantification relies upon reflected signal acquisition, which is significantly affected by surface roughness through photon scattering and collection artifacts. Additionally, while we have demonstrated the enhancement effect of hydrostatic pressure, other physical mechanisms inherent to hydrothermal vent mineral assemblages, including nanoscale effects, microcavity resonance and thermal gradient-induced self-focusing effect [1–3], could potentially improve upconversion efficiency by several orders of magnitude beyond our current values. Addressing these uncertainties will require both improved deep-sea spectroscopic techniques and refined characterization of the photon-generation capacities of hydrothermal minerals.

Out of the concerns about visible photon flux at hydrothermal vents, Conrad W. Mullineaux and Christopher D.P. Duffy proposed that cyanobacteria might adopt chemolithoautotrophic growth that is analogous to the cyanobacteria found in deep subsurface rocks from southern Spain [4]. However, by applying stringent criteria (retaining only genes covered by reads at ≥80% coverage), our analysis reveals that the Spanish subsurface cyanobacterial populations have lost some of the *cpc* genes and nearly all of the *cpe* genes encoding the phycocyanin and phycoerythrin of phycobilisomes, respectively (Supplementary Fig. S6 in Ref. [5]). Notably, over half of the key photosynthetic genes encoding photosystem I (PSI), photosystem II (PSII), electron transport chains and Cyt *b_6_f* were absent in sample T1_01. These genomic losses, combined with the absence of a chlorophyll autofluorescence signal in their deep subsurface rock samples, demonstrate the degeneration of the photosynthetic apparatus.

In striking contrast, cyanobacteria inhabiting high-temperature, chalcopyrite-rich hydrothermal vents retain complete oxygenic photosynthesis gene clusters (PSI, PSII and phycobilisomes). This genomic preservation provides strong evidence for persistent environmental selective pressure maintaining photosynthetic capacity in these extreme environments.

In environments lacking solar irradiation, minimal visible-light stimulation remains indispensable for cyanobacteria to retain their key oxygenic photosynthetic apparatus. The degenerate photosynthetic systems in Spanish subsurface strains arose precisely because of the total absence of light. The persistence of complete oxygenic photosynthetic gene clusters in cyanobacteria from high-temperature, chalcopyrite-rich hydrothermal vents strongly suggests that mineral-upconverted light exerts significant selective pressure to sustain those photosynthetic functions. Particularly, the upconversion emission from chalcopyrite in the blue–green band matches the absorption spectra of key pigments in cyanobacteria, such as Chl *a* (*λ_max_* ∼430 nm), phycourobilin (*λ_max_* ∼495 nm), phycoerythrobilin (*λ_max_* ∼560 nm) and phycoviolobilin (*λ_max_* ∼561 nm) [6], providing a plausible photic driver for this genomic conservation. Furthermore, the exclusively chemolithoautotrophic hypothesis cannot explain why vent cyanobacteria would maintain energetically costly photosynthetic complexes if light were irrelevant. Instead, we propose that mineral-mediated upconversion establishes a persistent niche, enabling phototrophic or potentially mixotrophic strategies. This model provides a coherent explanation for both the genomic evidence and the known metabolic flexibility of extremophilic cyanobacteria.

Finally, the potential for light emission from deep-sea hydrothermal vents to support oxygenic photosynthesis remains an open question that can only be resolved upon the successful isolation of cyanobacteria from these environments. Here, our metagenomic analyses (Supplementary Fig. S6 in Ref. [5]) exclude the possibility of far-red light-driven photosynthesis, but confirm the presence of phototrophic pathways adapted to visible light. These findings suggest that visible-light emission through frequency-doubling upconversion in sulfides could act as a selective force favoring photosynthetic microorganisms at hydrothermal vents. Given the presence of visible-light-harvesting systems at deep-sea hydrothermal vents and the metabolic flexibility of cyanobacteria, we propose that future research should prioritize: (i) precise *in situ* measurements of vent light flux, (ii) the isolation and cultivation of deep-sea cyanobacteria and (iii) investigation of metabolic activity enhancement under hydrothermal conditions (e.g. high temperature/pressure), in order to better understand metabolic adaptations to deep-sea hydrothermal vent environments with low photon availability. Technological advances in deep-sea exploration will be crucial to solving this enduring scientific puzzle.
